# Supplementary material for: Recalibrating probabilistic forecasts of epidemics
Source: PLoS Comput Biol. 2022 Dec 15;18(12):e1010771. doi: 10.1371/journal.pcbi.1010771 (PMC9799311; doi:10.1371/journal.pcbi.1010771)
Supplement: S1 Appendix — (PDF) [file pcbi.1010771.s001.pdf]

S1 Appendix

1 Examples of PIT Distributions

For illustration, we provide the densities of the PIT distributions for each of the 27 FluSight forecasters and four short-term targets, before recalibration (Fig S1) and after recalibration (Fig S2). The original forecasters' PIT distributions fall mostly into one of two categories: underconfident with a mode around 0.5, and overconfident with a minimum around 0.5 and peaks at 0 and 1. The outlier with a peak around 0.1 is the PIT distribution of the uniform forecaster. The recalibrated forecasters' PIT distributions are mostly flat, indicating that the PIT values are distributed nearly uniformly.

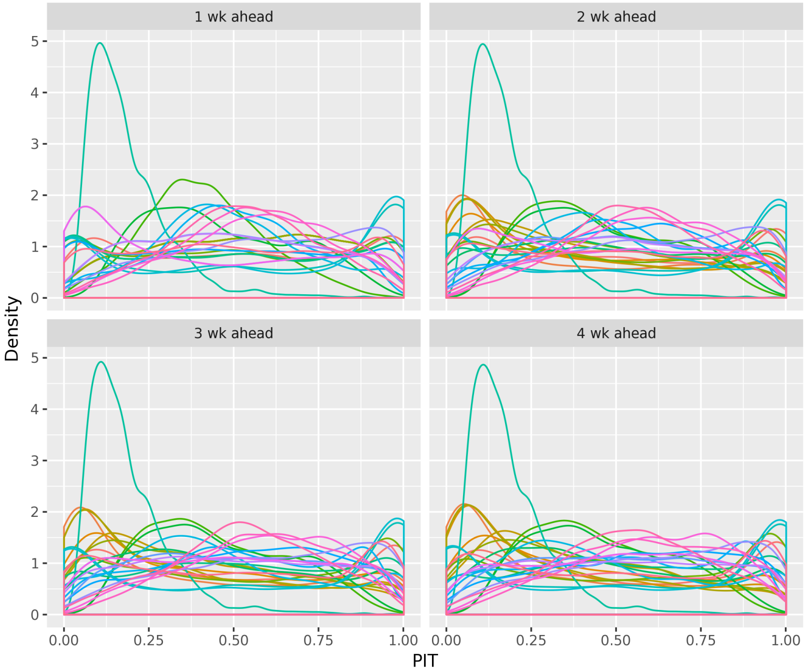

Fig S1. For each of the four short-term targets, the PIT distribution of each of the 27 FluSight Network component forecasters, before recalibration.

2 PIT Variance of Original and Recalibrated Forecasts

We show the variance of the PIT distributions of the original and recalibrated forecasts in Fig S3. The variance of the uniform distribution is $\frac{1}{12}$, and a forecaster whose PIT values have a variance of $\frac{1}{12}$ are referred to as *neutrally dispersed*. If the variance is greater than $\frac{1}{12}$, the forecaster is *underdispersed* ("overconfident"), and if the variance is less than $\frac{1}{12}$, the forecaster is *overdispersed* ("underconfident") [1]. Nearly all forecasters converge to a PIT variance close to $\frac{1}{12}$, and overconfident forecasters

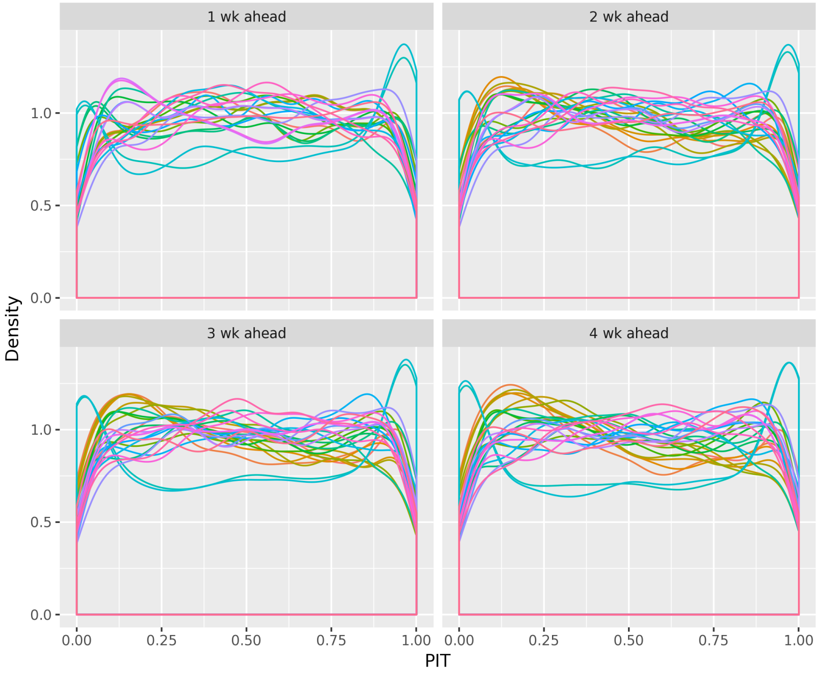

Fig S2. For each of the four short-term targets, the PIT distribution of each of the 27 FluSight Network component forecasters, after recalibration.

generally remain slightly overconfident, and underconfident forecasters generally remain slightly underconfident.

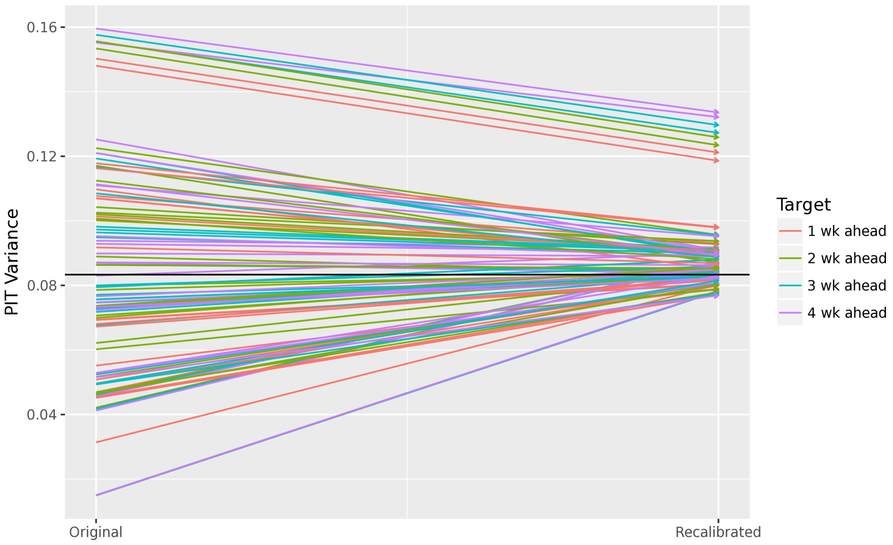

Fig S3. The variance of the PIT values before and after recalibration. In nearly all cases, the variance of overconfident forecasters decreases and the variance of underconfident forecasters increases. The variance of the uniform distribution is indicated by the black line at 1/12.

3 Recalibration Performance in a True Retrospective Setting

While there are many seasons available for recalibration training in the FluSight Challenge, this may not be the case for other epidemics. In Fig S4, we show how the component recalibration methods perform in a true retrospective setting, able to train on only past seasons. The parametric method improves performance with just one season of training data, and the nonparametric method improves performance after three seasons of training data.

Because influenza seasons can differ significantly, these results are subject to high variance and may not generalize. For example, in comparing this figure to Fig 8 in the main manuscript, the improvement in 2011 is substantially higher than the average improvement after one training season in Fig 8. Conversely, the improvement in 2018 is substantially lower than the average improvement after eight training seasons in Fig 8. We speculate that this is the reason that improvement decreases after 2014, despite an increase in available training data.

Note that for consistency, we only trained on available PIT values within a 3-week window on either side. In a real application with only one season available, the bias-variance tradeoff would likely favor a larger window and better performance.

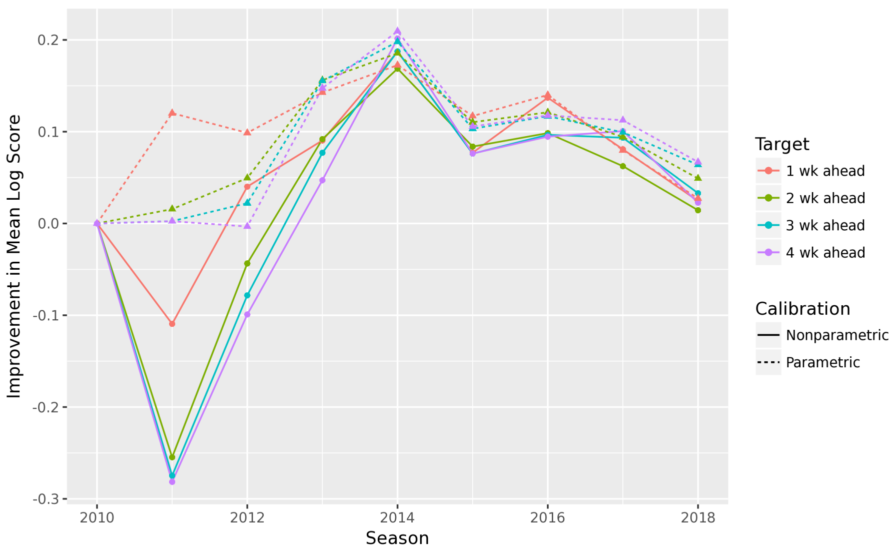

Fig S4. The improvement in mean log score for two recalibration methods, in a true retrospective setting. The parametric model results in an average increase in performance in the first season, and the nonparametric method in the third season.

4 Ensemble Weights

Our recalibration ensemble fits weights to three components: a parametric method, a nonparametric method, and a null method. In general, ensemble weights do not necessarily correlate with performance, and we find that to be the case here. We had

expected poor forecasters to have higher weights for the nonparametric and parametric methods than good forecasters, because they rely on calibration more strongly. However, the correlations between original forecaster performance and component weights are weak.

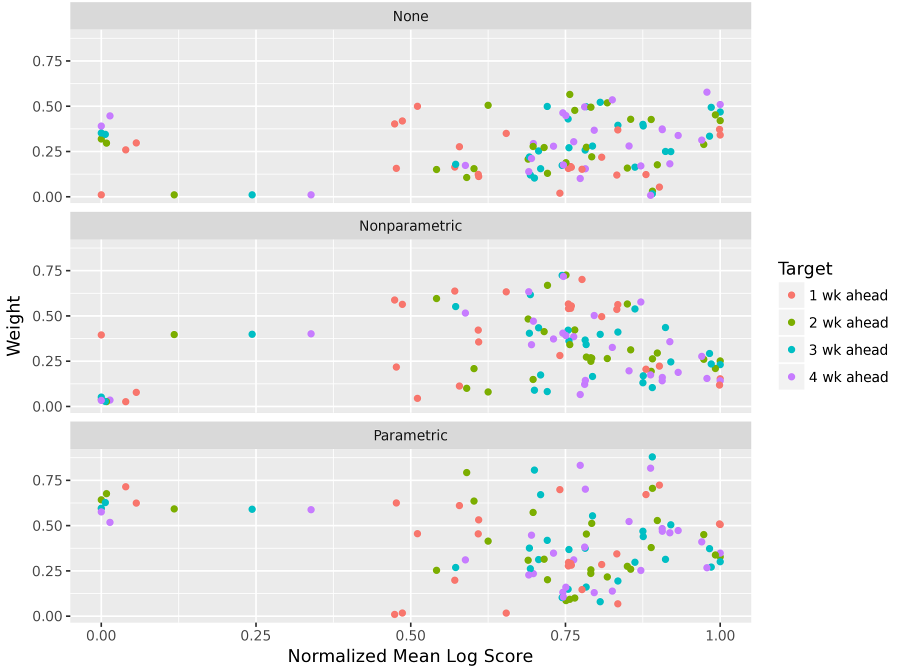

Fig S5. The ensemble weights for the three components, based on the mean log score of the original forecaster. Normalization is done by linearly mapping the scores to the [0,1] range, separately by target.

5 Improvement in Mean Log Score and PIT Entropy on Seasonal Targets

As mentioned in the discussion, forecasts of seasonal targets are difficult to recalibrate. After the season onset and peak have been observed, the true value is essentially determined, subject to data revisions. In such a case, a forecaster will place 100% of its mass in the correct bin, resulting in a PIT distribution which is a Dirac delta distribution $\delta(0.5)$. This forecaster has a PIT entropy of $-\infty$ but a perfect log score, which violates our previous assumption that improving the PIT entropy through recalibration will improve the log score.

In practice, there is a strong positive correlation between improvement in PIT entropy and improvement in mean log score, as shown in Fig S6. However, unlike the short-term targets, where the linear relationship had a slope of approximately 1, as theoretically expected, the slope for the seasonal targets is about 0.8. We suspect that the improvement in accuracy is not as large as theoretically expected because of forecast behavior at the end of the season.

However, a comparison of Fig S7 and Fig 7 in the main manuscript shows that recalibration on seasonal targets is much less effective than on short-term targets. No forecaster achieves a near-uniform PIT entropy. We believe this is because at the end of the season, the PIT distribution approaches $\delta(0.5)$, and the composition of any CDF

transform with a Dirac delta distribution will result in a Dirac delta distribution.

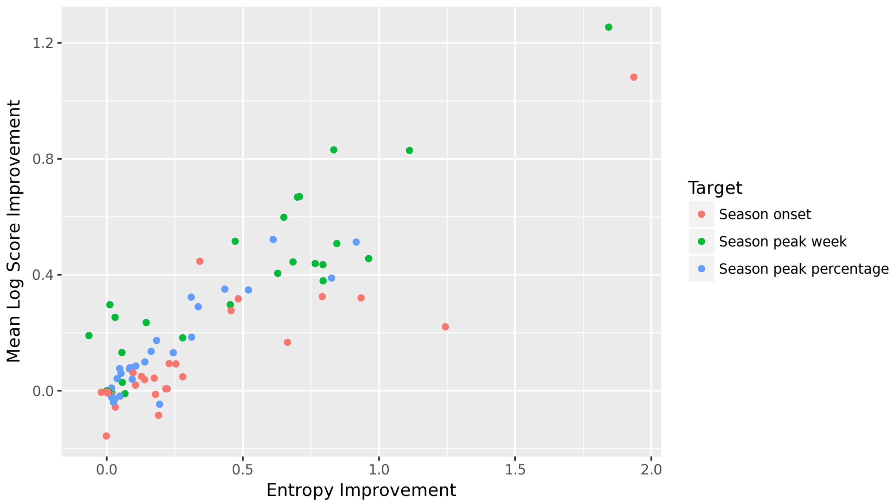

Fig S6. Improvement in mean log score versus improvement in PIT entropy for each of the 27 FluSight forecasters and seasonal targets. There is a clear linear trend between improvement in calibration and improvement in accuracy, although the slope is around 0.8, less than 1.

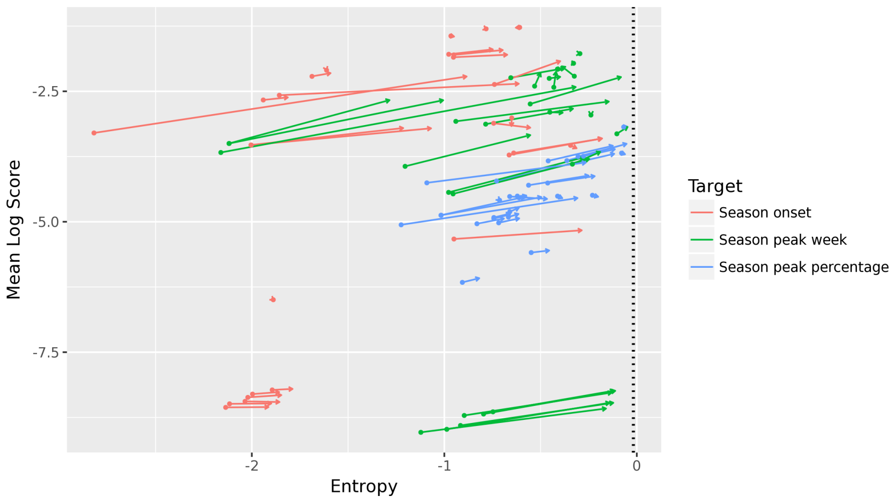

Fig S7. Entropy and mean log score before and after recalibration, for each of the 27 FluSight forecasters and seasonal targets. The tail of arrow represents a quantity before recalibration, and the head after recalibration. The dotted lines show the central 90% interval of the entropy of a comparably-sized sample of standard uniform random variables for comparison. Recalibration is much less effective for seasonal targets than for short-term targets (compare to Fig 7 in the main manuscript).

References

1. Gneiting T, Ranjan R. Combining predictive distributions. Electronic Journal of Statistics. 2013;7:1747–1782.
