# Supplementary material for: Recalibrating probabilistic forecasts of epidemics
Source: PLoS Comput Biol. 2022 Dec 15;18(12):e1010771. doi: 10.1371/journal.pcbi.1010771 (PMC9799311; doi:10.1371/journal.pcbi.1010771)
Supplement: Attachment — Submitted filename: RumackResponse.pdf [file pcbi.1010771.s002.pdf]

Recalibrating probabilistic forecasts of epidemics

Response to Reviewer Comments

We thank the editor, associate editor, and reviewers for their comments. Below, we respond to each comment individually. We also summarize the main changes to the manuscript here:

1. In Section 2.6, we revise the description of the seasonal component of the method to clarify that it is only relevant for forecasts of seasonal epidemics.
2. We add Section 3.3, which provides a sensitivity analysis with respect to the number of seasons available for training the recalibration methods.
3. We add a paragraph in the discussion that describes why we believe that this method will be useful in recalibrating forecasts of other epidemics, including those with limited training data and nonseasonality.
4. We add a Supporting Information file with additional plots in response to some of the minor points raised by the reviewers. These will be mentioned in the comments below.

1 Reviewer 1 Comments

Reviewer Summary: The authors present a novel technique for recalibating black-box forecasters that uses the distribution of PIT values from past forecasts to improve measures of calibration as well as log scores of future forecasts. They demonstrate the effectiveness of their approach by showing the improvement achieved for seasonal forecasts of influenza. Their proposed approach in a first step estimates PIT distributions (using both a parametric and a non-parametric method) and uses it to recalibrate forecasts. In a second step, they create a weighted ensemble of these two approaches and an unmodified forecast. To my knowledge this approach is novel and is one that I find genuinely exciting. I think the manuscript could be made even better by addressing a few points.

We thank the reviewer for their praise and constructive criticism.

1.1 Reviewer 1 Major Comments

Comment 1: First, the FluSight data set chosen to me feels like an idealised example, but this is not discussed in the paper. The authors use data that would not be available to researchers in many real-time settings, for example by using all other seasons, even those that lie in the future, to recalibrate forecasts for a given season. The data set is also very rich, including forecasts from the exact same models available across 9 seasons. In many settings, however, models evolve over time and it may not be possible to create retrospective forecasts. Diseases also may be non-seasonal or a disease may be seen for the first time in a given region (e.g. Ebola, Chikungunya in the Americas, COVID-19). I think it would be important to make these particular features of the example data clear and discuss in greater detail in which other settings the recalibration approach can be applied and where it may fail.

These are valid criticisms of the choice of the FluSight data. We decided to use data from future seasons because, under the assumption that each season is somewhat i.i.d., the improvement of recalibration in

the current FluSight real-time setting (with nine seasons to train on) will be closest to the improvement of recalibration trained on eight other seasons. Had we trained in a true retrospective approach, recalibration performance would be worse and not indicative of recalibration performance in the current FluSight real-time setting.

That being said, we agree with the reviewer that it would be useful to know how the recalibration method performs with less training data. We have included a new Figure 8 in the main paper which shows the performance of the nonparametric and parametric method when trained on $n \in \{1, 2, 4, 8\}$ seasons. We have also included the results from leave-future-out cross validation in the Supporting Information (Figure S4).

We agree with the reviewer that if models evolve over time and retrospective forecasts cannot be produced, recalibration will be much less effective, if effective at all. However, we believe that it is rare that recalibration will be harmed. Regarding model changes over time, it is usually straightforward to produce retrospective forecasts of the current model for a previous point in time. In any forecasting exercise, models are often chosen based on performance on a validation set, and this process is dependent on retrospective forecasts. If cross validation can be done for model selection, it can also be done for recalibration.

We appreciate the feedback regarding a wider class of diseases. Below, we reference the added text to the discussion section in which we address these points:

> We believe that this recalibration method can be applied to forecasting other diseases as well. Recalibrating influenza forecasts avoids challenges present in other forecasting environments, such as little training data, nonseasonality, and consistent forecasting models spanning many seasons. As shown in Figure 8, recalibration can improve performance with relatively little training data, although to be sure, applying this method will likely hurt performance where training data is severely lacking. In application to nonseasonal diseases, this method can easily be modified to use PIT values from the entire year, as opposed to the selective training used for influenza forecasts. Alternatively, selective training could be done not by calendar week but by some other feature(s) that differentiates a forecaster's behavior (e.g., whether cases are increasing or decreasing). The ensemble approach allows for the incorporation of multiple models trained on different historical forecasts, or even different recalibration methods altogether. Finally, even if a forecaster has been modified continuously over many years and previous performance is not indicative of current performance, recalibration can be trained on retrospective forecasts produced by the current forecaster.

Comment 2: Secondly, I think Figure 3 and Figure should be made more consistent and captions should to be more detailed, as it is sometimes not clear what the figure shows. In particular:

- Figure 3: I'm not sure I exactly understand what was done. Why was the log score of the most accurate forecaster chosen, rather than say the average of all forecasters? What forecast horizon was used? Maybe it would be helpful to clearly separate k and 'window size' as at least to me they were confusing at first. Do I understand correctly, that k = 10 means 10 weeks prior + 10 weeks past the week, so the window is actually 20 weeks? when k = 10, how do you deal with the forecasts in the first 10 weeks (or the last ones). Is the window shortened for these, or are they omitted from scoring? In addition: maybe it would be interesting to extend the shown window size even further, until we actually see performance degrade.

- Figure 4: It is slightly confusing that the non-parametric method performs about equally well for 2-, 3-, and 4-week-ahead forecasts in this plot. Just looking at Figure 3, one would expect a negative effect for 4-week-ahead forecasts and a 3-week window size. I assume this is because Figure 4 uses all 27 forecasters and Figure 3 only the best one? To me this is confusing and it would be helpful if the Figures were more consistent.

We have updated Figure 3 to use the average of all forecasters, and the results of Figures 3 and 4 are now in agreement. Additionally, we have updated the text to make the relationship between k and window size clearer (the reviewer's reading was correct) and also clarified that the window is shortened for the forecasts made in the beginning or end of the season.

Comment 3: Thirdly, the code, as of now, is not very accessible. It currently sits on a branch of a much greater repository and it is unclear a) whether this code is meant to stay there indefinitely, b) how the code ties in with the greater context of the repository (what is required for the recalibration and what is not) and c) how someone could use the code for their own work. I think it would greatly enhance accessibility of the code if the authors could a) move it to a separate repository (or maybe a subfolder of the main repository), rather than just a branch of an existing repository, b) document the code and its functions in greater detail and c) provide some more explanations on how to run the code.

Initially, the recalibration code was fully integrated in our influenza forecasting system, but was not efficiently modularized. When this manuscript was first submitted, our plan was to move the code to its own repository and strip away the bulk of the repository, which was unnecessary for the recalibration code. This has now been completed (https://github.com/rumackaaron/recalibration) and the reference in the manuscript has been updated. We have also added documentation to the calibration code.

1.2 Reviewer 1 Minor Comments

Comment 1: Introduction: Maybe it would be a good idea to mention the forecasting paradigm to maximise sharpness subject to calibration (see Gneiting et al., Probabilistic forecasts, calibration and sharpness, 2007)?
Although Gneiting uses this paradigm, we do not feel that it is relevant to our approach. That phrasing seems to imply that calibration is a simple task whereas sharpness is a difficult one, and a forecaster should try to create sharp forecasts so long as they are calibrated. Our approach is in some ways the opposite: calibration is difficult for a forecaster to achieve, and we provide a method to maximize calibration subject to the forecaster's initial sharpness.

Comment 2: Line 10: If calibration is one aspect, which are the other two? This is explained in the discussion, but maybe could also be included here.
We have added uncertainty and resolution to the relevant sentence.

Comment 3: Line 58: I asked myself what the the added complexity was if CDFs were not strictly increasing. Maybe either remove "for simplicity" or add a short explanation of why this is necessary?
We replaced "for simplicity" with "as a regularity condition." If the CDFs are not strictly increasing, then the forecast PDFs and PIT PDF can be 0, which is problematic in the equations in Section 2.1.

Comment 4: Line 59: I'm not sure why the Brier Score which is usually associated with binary forecasts is mentioned here. Maybe this could be made a little more clear (or removed).
We replaced the reference to the Brier score with the quadratic score, and we thank the reviewer for pointing out that we used the wrong name for the continuous scoring rule.

Comment 5: A few lines below line 60 (there are no line numbers here unfortunately): The observation was previously called x_i, (and y_i was the random variable). It is maybe slightly confusing that the observation is now called y_i, where before the observation was x_i.
We apologize for the confusion, and we have revised the text for clarity. We always use y_i to refer to the observation drawn from h_i. We use x_i only to denote information used as input to a forecaster.

Comment 6: Line 70: Maybe mention that a uniform PIT is a necessary, but not a sufficient condition for probabilistic calibration. Gneiting et al. (Probabilistic forecasts, calibration and sharpness, 2007) and Hamill (Interpretation of Rank Histograms for Verifying Ensemble Forecasts, 2001) mention examples of forecasters who are mis-calibrated, but have uniform PIT histograms.
Gneiting and Hamill give examples of forecasters which are *not ideal* and have uniform PIT histograms. For

a subset of forecasts, the forecasters are miscalibrated, but over the entire set, these forecasters are indeed probabilistically calibrated. As we demonstrate in the equation above line 68, the expected PIT CDF of a probabilistically calibrated forecaster is the identity, meaning that the PIT distribution is uniform. This is stated elsewhere as well (see Gneiting and Ranjan, Combining predictive distributions, 2013, Definition 2.6b).

Comment 7: Equation 2: Again x_i and y_i are used differently than before
See response to comment 5.

Comment 8: Line 144 "too conservative" is slightly ambiguous. What it means is that forecasters don't adapt to trends quickly enough, but it could also easily be understood as "forecasts are to wide and uncertain"
We agree that the phrase "too conservative" is ambiguous and is superfluous anyway, so we have removed it.

Comment 9: I unfortunately did not really understand the sentence in lines 178-180. Maybe the exact format could be worded more clearly?
We have reworded the format of the forecasts for clarity. The text now reads:

> Each forecast is discretized over predetermined bins, forming a histogram distribution. For the season onset and season peak week targets, the width of each bin is one week, and for the other targets, the width of each bin is 0.1% wILI.

Comment 10: line 185: In applied setting, would't it make more sense to use leave-future-out CV? Currently this is using data that would not have been available to researchers making forecast at the time. This ties into the point made in the summary above: I would really like to know what performance is in less idealised circumstances.
We agree that it is important to do a sensitivity analysis, and we have done so. As mentioned above, we added a new Figure 8 which plots performance over varying numbers of training seasons and a plot showing performance of leave-future-out CV in Figure S4.

Comment 11: I'm not entirely sure I understood the full algorithm explained from line 187 on. What is confusing to me is that seasons are denoted with r and s, and weeks with i, but then later on (e.g. line 193), seasons are also denoted with i. I am also not sure I understood the difference between step 1 and step 3.
We thank the reviewer for catching the typo of indexing seasons with i. We also clarified that the forecasts in step 1 are only used for calculating the weights in step 2, whereas the forecasts in step 3 are the components of the recalibrated ensemble.

Comment 12: Maybe it would be helpful to point out whether the weeks i in the seasons calendar correspond to calendar weeks, or are they relative to something?
In practice, the weeks i are calendar weeks. Because the influenza season begins in the fall, the 35th week of the calendar year is often counted as the first in the season. In other applications, the indexing can vary and the weeks do not necessarily have to be from Sunday to Saturday. Because of this, we felt that it would be distracting to include this in the algorithm description.

Comment 13: 218 - 19: This sentence implies that the amount of improvement is close between the two, but rather I understand the proportion of improved models is close.
We thank the reviewer for catching this. In Figure 6, the amount of improvement is close, but Figure 5 shows that the proportion of models is close.

2 Reviewer 2 Comments

2.1 Reviewer 2 Major Issues

The paper gives a nice discussion (Sec 2) of the too-often-overlooked recalibration issue for influenza forecasting.

Relative to the 4-step recipe for the recalibration approach in Section 3, there appear to be implicit assumptions that should hold for the approach to be reasonable. For example, Step 1 defines a set of relevant (essentially exchangeable?) forecast errors for a "current" forecast, and it relies strongly on a seasonal structure. Despite certain claims (as in Sec. 2.6 and "the seasonal nature of epidemic forecasting"), not all highly communicable diseases are intrinsically seasonal, let alone do they have ample seasonal data available for recalibration. Examples: SARS, ebola, many animal diseases, and to this point in time, covid. The seasonal assumption clearly applies to influenza, but may not generalize easily to other forecasting environments.

We thank the reviewer for their positive comments and constructive criticism. The recalibration approach does assume that a forecaster's errors, as measured by the PIT values, come from the same distribution in both the training set and the test set. Our algorithm for influenza forecast recalibration relies strongly on a seasonal structure, but this seasonal step is simple to remove for nonseasonal diseases by estimating the PIT CDF from all available PIT values. We reference the paragraph added to the discussion section of the manuscript, as quoted above in response to Reviewer 1's first major comment.

Comment 2: Next, it appears implicit that a forecaster's prediction errors are fundamentally static. Stated less charitably relative to your FluSight example, were all forecasters so oblivious to their forecasting performance over the multi-year forecasting time frame that they never learned anything that helped them improve their modeling? Do the results of your analysis have no value to them beyond modifying prediction intervals after the fact? At a minimum, shouldn't this static assumption (i.e., that forecasters can never learn nor modify their behavior) be somehow confirmed before the recalibration approach is routinely applied? As in the previous paragraph, the assumption appears to give good results for influenza, but may(?) not generalize to other dynamic forecasting environments.

The recalibration method does also assume that the forecaster's prediction errors are static. Each forecaster in the FluSight ensemble submitted nine seasons worth of forecasts from the same exact model and code, so in this case, we know that the forecasters did not change their algorithm from one year to the next based on previous mistakes. In dynamic forecasting environments, recalibration would be trained on retrospective forecasts made from exactly the same model as opposed to a previous model. We have clarified this in Section 3, with the added text quoted below:

> The retrospective forecasts were produced at the same time, with each forecaster using the same method for all seasons. Had the forecaster modified its algorithm from season to season, the previous forecast performance would not be predictive of future forecast performance, violating the assumptions behind this recalibration method.

Comment 3: A suggestion is that underlying assumptions such as exchangeability and static forecasts be made explicit. Further, if the authors truly feel that their approach works as well for other infectious diseases as it does for influenza, some justification of this claim together with concrete examples would be a welcome addition to the paper. Otherwise, the emphasis of the paper (as well as its title) should be modified to reflect the influenza-centric nature of the approach.

We have added these assumptions explicitly in the paper, and we added a paragraph in the discussion (quoted above) to justify why we believe this approach will work well for other infectious diseases.

2.2 Reviewer 2 Minor Issues

Comment 1: I was struck by the comment on p.1 in the summary that "epidemic forecasting is a relatively new field". Different points of view are not hard to find, e.g., "failure in epidemic forecasting is an old problem" (Ioannidis, Cripps, and Tanner; International Journal of Forecasting, 2021, Sec. 1). Indeed, the long/undistinguished history of epidemic forecasting is actually good motivation for your work. Or maybe there's some confusion over what your use of the word "new" means?

Our statement was made relative to weather forecasting and econometric forecasting, which are the applications of recalibration in the bulk of relevant publications. Given the confusion, we have simply omitted the phrase.

I was disappointed at the lack of an overall discussion of the optimized weight values $\{w_i\}$ for each ILI forecaster/target. What do the optimized weights imply about the three components of the ensemble (Sec 2.5) and/or about the individual forecasters? Could some detail be added on this subject?

In general, ensemble weights are not indicative of performance, so we originally decided to not discuss this in the manuscript. However, we added a paragraph to Section 2.5, quoted below, and added a plot to the Supporting Information (Figure S5).

> A component's weight in the ensemble is not necessarily proportional to that component's performance. For example, if the two best components are very similar to each other, one may have a very small weight because that component's information is effectively represented by the other component.

I liked the comment "recalibration only improved the performance of about three-quarters of the forecasters" on seasonal targets as it provided some insight to the analysis. Unfortunately, the discussion of this issue in the last paragraph of Sec. 4 did little to distinguish which types of forecasters gain by using the approach and which types don't. Was it that one-quarter of FluSight forecasters were well calibrated to begin with, so that no major improvement was possible? Did the aforementioned one-quarter of forecasters modify their predictive model over time, invalidating the implicit assumptions? Or ...? Providing more intuition as to when the approach works well (and when it doesn't) would be a nice addition to the paper.

Seasonal targets are difficult to reason about, because at the end of the season, the true value has already been observed and the prediction task is trivial. In such a case, a forecaster will place 100% of its mass in the correct bin, resulting in a PIT distribution which is a Dirac delta distribution $\delta(0.5)$. This forecaster has a PIT entropy of $-\infty$ but a perfect log score, which violates our previous assumption that improving the PIT entropy will improve the log score. Because of this paradox, we are reluctant to make claims about why recalibration might help a certain type of forecaster on seasonal targets.

We have added two plots to the Supporting Information (Figures S6 and S7). The first shows that there is a strong positive correlation between PIT entropy improvement and mean log score improvement for seasonal targets, just as we find for the short-term targets. We find that the quarter of forecasters that do not improve accuracy also see a small improvement in PIT entropy, if any. The second plot shows that no recalibrated forecaster achieves a near-uniform PIT distribution for seasonal targets, and that the original forecaster's accuracy and entropy are not strong predictors of how recalibration will improve a forecaster.

Most influenza forecasters have a reputation of being overconfident, with U-shaped PIT curves. Does residual overconfidence still exist for your recalibrated forecasts, though to a much lesser degree? Is the approach mainly a bias correction activity?

We thank the reviewer for this question. We have added Figure S3 that describes overconfidence, as quantified by the variance of the PIT values before and after recalibration. Gneiting and Ranjan (Combining predictive distributions, 2013) use the PIT variance to define a forecaster as over- or underdispersed. It turns out that the reputation of overconfidence is unjustified, as about half of the forecasters are overdispersed and half are underdispersed. The PIT variance of the recalibrated forecasters shrinks towards $\frac{1}{12}$, the variance of the uniform distribution.

Although recalibration brings the PIT variance towards $\frac{1}{12}$, recalibration is more than just bias correction.

The nonparametric method can correct a PIT distribution of any shape, given sufficient training data. Although the parametric method is less flexible, in a case where the PIT distribution cannot be approximated by a beta distribution, the nonparametric method should receive a larger weight in the ensemble.

Lastly, could the paper could note that the approach can be easily adapted to incorporate other recalibration methods beyond just those considered in the paper.
We thank the reviewer for this suggestion and we have noted this in the discussion section.
