# Supplementary material for: Recalibrating probabilistic forecasts of epidemics
Source: PLoS Comput Biol. 2022 Dec 15;18(12):e1010771. doi: 10.1371/journal.pcbi.1010771 (PMC9799311; doi:10.1371/journal.pcbi.1010771)
Supplement: Attachment — Submitted filename: Rumack_Response.pdf [file pcbi.1010771.s003.pdf]

Recalibrating probabilistic forecasts of epidemics

Response to Reviewer Comments

We thank the editor, associate editor, and reviewers for their comments. Below, we respond to each comment individually. The main change to the manuscript is in the discussion section, where we discuss in greater depth the strengths and weaknesses in applying our recalibration method to other diseases.

1 Reviewer 1 Comments

Thank you very much for addressing our comments. I would be very happy to see this work published.

We thank the reviewer for their approval and previous comments.

2 Reviewer 2 Comments

Comment 1: In my previous review, I asked you to give specific, concrete examples of seasonal diseases (besides influenza) for which currently available public health data allow for the use of your methodology. I was disappointed that you couldn't/wouldn't provide a single such example. The paper would be much improved if its sweeping generalizations about applicability (such as lines 296-297) were accompanied by some detailed supporting evidence.

We apologize for the previous omission, and we have included dengue and Aedes mosquito counts to the paper. Both of these have up to 10+ years of data in many locations, have a seasonal component, and have been the subject of previous CDC forecasting competitions.

Comment 2: In any case, the approach has potential benefit for influenza forecasting. That some of these predictive models have been used for as long as they have and still aren't well calibrated is somewhat embarrassing to the epi community – especially in light of recent high-profile problems with poor/no uncertainty estimates for COVID-19 forecasts (see the reference cited in my previous review) and the history of other epidemiological probabilistic assessments, such as questionable confidence intervals for mechanistic model parameters (see, e.g., general work by Ioannidis, Tong, and others). Recalibration of probabilistic statements is topical and important.

Comment 3: Also on a positive note, the basis for the approach is more clearly presented in the revision. Adding discussion in Section 3.3 on varying amounts of training data is a nice touch, although the text is misleadingly one-sided. The good news, as is well summarized, is that there is only modest loss of precision with limited training data so long as the modeling assumptions are valid. The bad news, however, needs attention: having limited training data means having limited ability to meaningfully check the validity of those assumptions in the first place, which undermines "robustness" (line 247) and could easily lead to poor results from the approach.

We have limited the claim in Section 3.3 to state only that recalibration was successful with limited training data on *FluSight Challenge* forecasts. We also add the following paragraph in the Discussion section:

> A lack of training data is a more difficult problem to solve. An obvious problem of limited training data is the variance in estimating $\hat{G}$, but an additional challenge is that it is difficult to confirm our assumption that the PIT distribution is stationary over time. If we cannot detect that the PIT distribution changes over time, we will make inappropriate "corrections" to the forecasts that could harm calibration and accuracy. In practice, recalibration improved performance of the FluSight Challenge forecasts with relatively little training data, as shown in Figure 8. In less well-behaved applications, however, performance could decrease.

Comment 4: Specifically, the stationarity assumption (lines 148-149) that the PIT distribution is the same for the training and test sets should not be taken lightly, especially for nonseasonal diseases. Consider this assumption relative to the most prominent nonseasonal disease at the moment, COVID-19, where the PIT distribution evolved over time for a variety of reasons. This type of nonstationarity can be circumvented by using only the most current data for recalibration, similar to the rationale in your lines 156-164 (aside: such an approach has recently been detailed – Picard and Osthus, medRxiv 2022; even then, daily data might be required for true success in practice). Although the COVID-19 paradigm is nearly antithetic to the static-seasonal-disease, static-forecast-model paradigm you focus on, it emphasizes the importance of having adequate training data to check modeling assumptions.

These are good points, and we have added the following paragraph to discuss them:

> In application to nonseasonal diseases, such as COVID-19 (currently), this method can easily be modified to use all available PIT values, as opposed to the selective training used for influenza forecasts. Alternatively, selective training could be done not by calendar week but by some other feature(s) that differentiates a forecaster's behavior (e.g., whether cases are increasing or decreasing). While this allows for a flexible approach to recalibrate a variety of seasonal and nonseasonal diseases, this may be difficult to implement effectively in practice. In other cases where the PIT distribution changes slowly over time, training could be done only on the most recent forecasts to improve the estimate of $\hat{G}$. This selective training approach has been successful in recalibrating COVID-19 forecasts [?]. The ensemble approach allows for the incorporation of multiple models trained on different historical forecasts, or even different recalibration methods altogether.

Comment 5: The added paragraph (lines 296-311) on nonseasonal diseases is very weak. Readers will immediately wonder: exactly which nonseasonal diseases are you talking about? What disease-specific data sets exist for forecasting, now or in the foreseeable future? What's so special about an "entire year" (line 303) for a disease that doesn't care about the calendar? Because these questions aren't addressed, readers will find this paragraph to be exasperatingly devoid of any substantive details. There's a case to be made for your assertions, but you're not making it. A related point: the paper's approach is somewhat ahead of its time, awaiting a world where timely, standardized, multi-year, and easily accessible training data are available for all seasonal and nonseasonal diseases of interest. Admittedly, public health and biosurveillance data are moving (very slowly) in this direction, but they're nowhere near that state now.

We expanded this paragraph into three paragraphs to provide more detail in examples and more depth in analysis. We mention COVID-19, dengue, and the Aedes mosquito as potential epidemiological forecasting targets. We also changed the wording from "entire year" to "all available PIT values." We thank the reviewer for their feedback.
